# A bibliometric analysis of Mediterranean diet on cancer from 2012 to 2021

**DOI:** 10.3389/fnut.2023.1128432

**Published:** 2023-02-08

**Authors:** Yafeng Liu, Jibin Lu

**Affiliations:** Department of Thoracic Surgery, Shengjing Hospital of China Medical University, Shenyang, China

**Keywords:** Mediterranean diet, cancer, bibliometric analysis, VOSviewer, CiteSpace

## Abstract

**Background:**

Numerous studies have demonstrated the value of the Mediterranean diet (MD) as a nutritious eating regimen for lowering the risk of cancer. This study aims to discuss the research patterns, existing state, and possible hotspots in implementing the MD for the prevention and treatment of cancer using bibliometrics.

**Methods:**

The Web of Science Core Collection (WoSCC) was searched for articles on cancer that were related to the MD. CiteSpace, VOSviewer, Microsoft Excel 2019, and R software were utilized for bibliometric analysis and data visualization.

**Results:**

There were 1,415 articles and reviews published from 2012 to 2021. Annual publication volume showed a continuous upward trend. Italy and Harvard University were the country and institution, respectively, with the highest number of publications on this topic. Nutrients ranked first in the number of documents, number of citations, and the *H*-index. James R. Hebert was the most productive writer, and Antonia Trichopoulou was the most co-cited author. “Alcohol consumption,” “oleic acid,” and “low density lipoprotein” were keywords used in earlier publications, while more recent hotspots focused on “gut microbiota,” “older adult,” and “polyphenol.”

**Conclusion:**

Over the past decade, research on the MD in the field of cancer has received increasing attention. To improve the level of evidence for the beneficial effects of the MD on a range of cancers, more research on molecular mechanisms and better clinical studies are required.

## 1. Introduction

Cancer is one of the leading causes of death worldwide, accounting for roughly 10 million deaths in 2020, or nearly one-sixth of all deaths. By 2040, there are expected to be 28.4 million instances of cancer worldwide (1, 2), accounting for an increasing impact and burden of cancer on public health at a global scale (3). Numerous studies have demonstrated that people are becoming increasingly aware of the positive effects of diet on cancer (4, 5). The Mediterranean diet (MD) was first proposed by Keys (6), who later identified that the monounsaturated fatty acids in the MD are effective in lowering mortality due to coronary heart disease. After decades of development, the MD has become defined as a comprehensive lifestyle that includes daily physical activity and that is based on a diet that includes a higher intake of whole grains, vegetables, and fruits. The MD includes olive oil as the main source of fat and fish and white meat as the main sources of protein. It also encourages the consumption of nuts and a small amount of red wine (7). Studies have become increasingly concentrated on the beneficial effects of the MD on cancer (8). The beneficial effects of the MD on breast cancer (BC) (9, 10), colorectal cancer (CRC) (11, 12), lung cancer (13, 14), prostate cancer (15–17), and other cancers have been confirmed.

American bibliographer Pritchard (18) originally introduced the concept of bibliometrics in 1969. Bibliometrics uses philological, mathematical, and statistical methods to quantitatively investigate the macroscopic rules of literature and can swiftly establish the current state and future potential development trends and hotspots of a research field by processing information such as countries/regions, institutions, journals, authors, references, and keywords (19).

Despite the large number of studies on the impact of the MD on cancer, the general developmental process, current state, hotspots, and future trends in this field are unknown. This study carefully synthesizes pertinent research over the last 10 years using bibliometrics to correctly depict and analyze the entire breath of research on the MD in the field of cancer.

## 2. Materials and methods

### 2.1. Data source and search strategy

**FIGURE 1:**
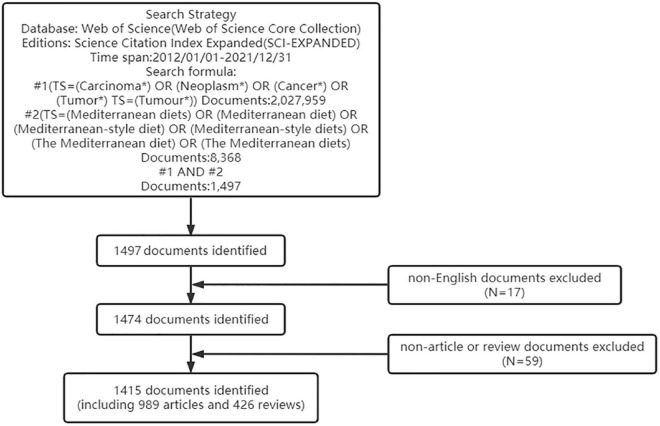
Search strategy and data filtering process.

Data was obtained from the Science Citation Index Expanded (SCI-EXPANDED) edition in the Web of Science Core Collection (WoSCC). To avoid errors caused by WoS database upgrades, all data searches and exports were performed on 7 December 2022. The search strategy was as follows: #1 Topic = (cancer*) OR (its synonyms); #2: Topic = (“MD”) OR (its synonyms); and incorporation of #1 AND #2. From 2012 to 2021, the language was limited to English. Article type was limited to original research and review articles. The detailed search strategy and data filtering process are shown in Figure 1.

### 2.2. Data analysis, visualization, and analysis index

Full records and cited references were downloaded for all documents obtained from WoSCC, which were received in TXT or BibTeX format. The bibliometrix package (20) in the R software (version 4.1.0) was used for extraction and statistical analysis of data (e.g., count/citation, title, abstract, publication year, source, author, country/region, and keyword). Microsoft Excel 2019 was used for data statistics that examined the volume of annual publications and their trends.

VOSviewer and CiteSpace are commonly used bibliometric and visualization software platforms (21, 22). VOSviewer (version 1.6.18) (23) is utilized for visual analysis of co-occurrence networks of countries/regions and institutions. CiteSpace (5.8.R3) (24) was developed by Professor Chaomei Chen and was originally designed for co-citation analysis and later extended to a variety of other functions. CiteSpace was employed for author and reference co-citation analysis, author co-authorship network analysis and visualization, keyword-related analysis and visualization, and dual-map overlap analysis of journals (25). Additionally, the R software circlize package (26) and ComplexHeatmap package (27) were used to partially show the country/region distribution of publications and collaborations. The number of documents or citations is indicated by the node size in CiteSpace and VOSviewer, with a larger node indicating more publications or citations. Relationships of collaboration, co-occurrence, or co-citation are represented by lines connecting the nodes. In CiteSpace, different node colors indicate different years; circles with a range of colors from the inside to the outside represent the years 2012 to 2021. The outermost purple ring indicates that the node has a very high centrality, which is usually regarded as a turning point between distinct yields. Impact factor (IF) and quartiles were obtained from the Journal Citation Report (JCR) 2021 and Quartile List as significant indicators of the study’s scientific worth. The *H*-index (28) was defined as the number of citations of at least *h* papers greater than *h* by an independent individual. It is used as an effective indicator to evaluate the academic influence for assessing both the quantity and quality of independent individuals such as countries/regions, institutions, journals, and authors.

## 3. Results

### 3.1. Annual publication volume and trend

**FIGURE 2:**
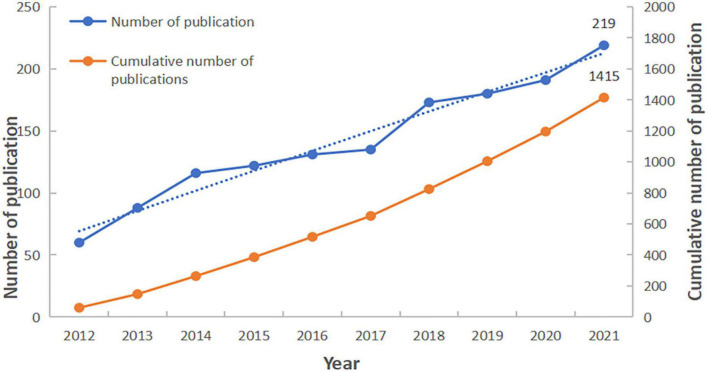
The annual volume and trend of publications on the Mediterranean diet (MD) on cancer.

Our search strategy identified a total of 1,415 papers, including 989 articles (68.99%) and 426 reviews (30.11%), related to the MD and cancer. The annual publication volume and trends in the field of the MD on cancer are illustrated in Figure 2. It is clear that over the past 10 years, the number of publications in this field has gradually increased, rising from 69 in 2012 to 219 in 2021. The overall trend in this area continues to rise, demonstrating that research on the effect of MD on cancer is at a stage of sustained positive development, and there will likely be a significant number of relevant studies in the future.

### 3.2. Distribution of countries/regions and institutions

**FIGURE 3:**
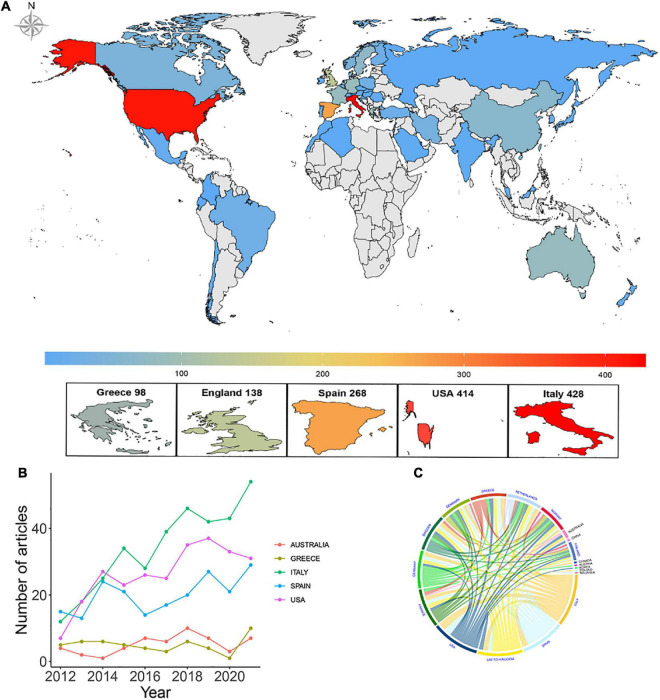
**(A)** Geographical distribution of the countries/regions in terms of publications. **(B)** The trends of publications by top five countries from 2012 to 2021. **(C)** The chord diagram of cooperation among countries/regions.

**FIGURE 4:**
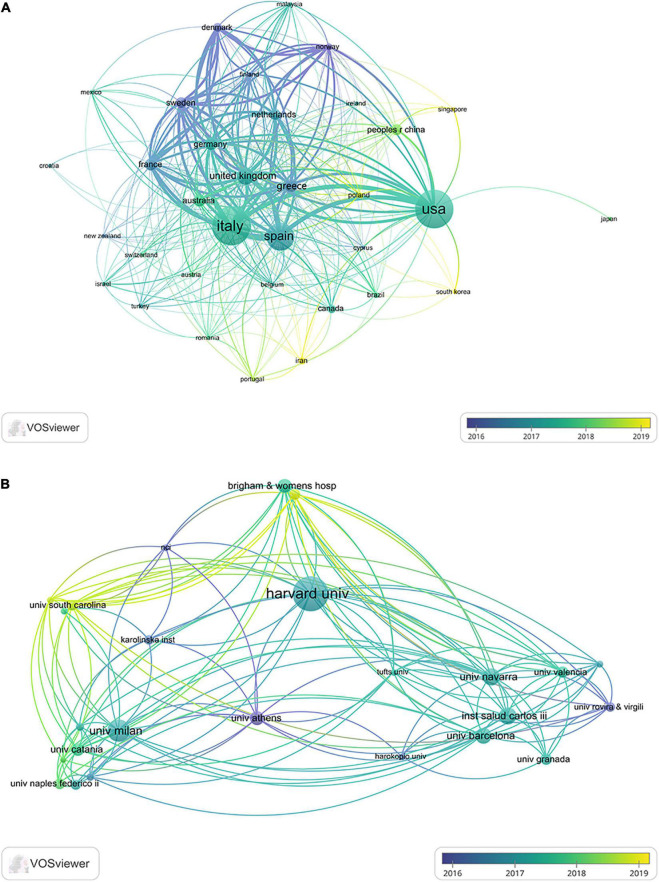
The co-authorship network diagram of panel **(A)** the countries/regions and **(B)** institutions. The size of the nodes represents the number of publications, the lines represent cooperation, and the color of the nodes represents the time.

A total of 77 different countries and 2,123 institutions participated in the study of the effects of the MD on cancer. Table 1 lists the top 10 countries/regions for scientific research production. Most publications were produced in Italy (428), followed by the United States (414), Spain (268), the United Kingdom (138), and Greece (98). Figure 3A displays the geographical distribution of publications by countries/regions. The top five countries/regions’ trajectory from 2012 to 2021 is shown in Figure 3B. Italy had the highest annual output and the fastest growth, while the United States and Spain have consistently maintained high output levels. Figure 3C depicts the cooperation between countries/regions. Italy was the country with the most active international cooperation, and the most common cooperation occurred between Italy and the United States. Figure 4A displays the network of international cooperation between countries/regions and the average publication time. Iran, Singapore, and the Republic of Korea have produced publications in this field in recent years (yellow nodes). Table 1 lists the top 10 research institutions by production, with Harvard University in the United States ranking first, followed by the University of Milan, Barcelona University, University of Navarre, and Granada University. With four institutions each in Italy and Spain, Italy and Spain housed the most institutions among the top 10 research institutions researching the MD and cancer. Co-authorship between institutions and the average publication time of research is shown in Figure 4B. Harvard University was the hub of inter-agency cooperation, and the University of South Carolina has published some studies in this field most recently.

**TABLE 1:** Top 10 productive countries/regions and top 10 productive institutions related to the Mediterranean diet (MD) on cancer.

Rank	Country/region	Count	Percentage (*N*/1,415)	Rank	Institution	Count	Location
1	Italy	428	30.25	1	Harvard University	229	USA
2	USA	414	29.26	2	University of Milan	112	Italy
3	Spain	268	18.94	3	Barcelona University	100	Spain
4	England	138	9.75	4	University of Navarre	100	Spain
5	Greece	98	6.93	5	Granada University	96	Spain
6	Germany	90	6.36	6	Carlos III Health Institute	92	Spain
7	Australia	83	5.87	7	University of Catania	89	Italy
8	France	83	5.87	8	University of Athens	80	Greece
9	Sweden	72	5.09	9	University of Florence	75	Italy
10	Netherlands	67	4.73	10	University of Naples Federico II	72	Italy

### 3.3. Journals

**FIGURE 5:**
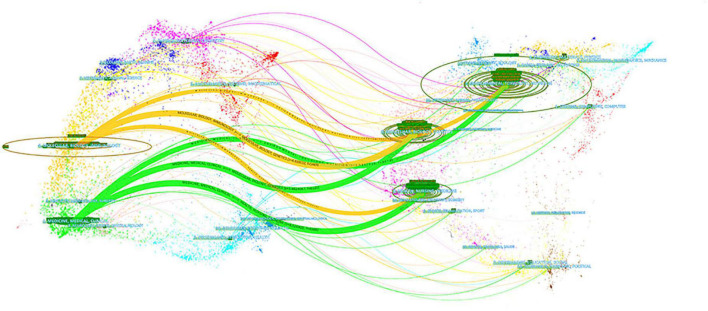
A dual-map overlap of journals of Mediterranean diet (MD) on cancer. The left is the cited journal and the right is the cited journal, the longer transverse diameter of the ellipse represents more publications in the corresponding journal.

The 1,415 papers included were published in 450 different journals. The top 10 journals by publication volume are shown in Table 2, with *Nutrients* having the greatest volume with 158 papers (11.17%), followed by the *British Journal of Nutrition* (44, 3.11%), *European Journal of Nutrition* (41, 2.90%), *American Journal of Clinical Nutrition* (34, 2.40%), and *Journal of Nutrition* (33, 2.33%). When ranked by the number of citations, the top five journals were *Nutrients* (4,927), *Journal of Nutrition* (1,798), *British Journal of Nutrition* (1,795), *International Journal of Molecular Sciences* (1,748), and *American Journal of Clinical Nutrition* (1,716). According to the magnitude of the *H*-index, the top journal was *Nutrients* (37), and the *British Journal of Nutrition* (23) and *American Journal of Clinical Nutrition* (23) were tied for second place. A total of 80% of the top 10 journals were in Q1 and Q2, with 40% in the United States and 30% in Switzerland. These publications ranged in IF (2021) from 2.816 to 8.472. Figure 5 shows the dual-map overlap of journals with publications on the MD and cancer. The distribution of subjects between citing journals (left part) and cited journals (right part) is illustrated in Figure 5. As shown in Figure 5, the three most significant relationship bands demonstrate that the pertinent research in this field was mainly published in journals belonging to Medicine/Medical/Clinical or Molecular/Biology/Immunology subjects. Furthermore, these studies were mainly based on research published in journals belonging to Molecular/Biology/Genetic or Health/Nursing/Medicine subjects.

**TABLE 2:** Top 10 productive journals related to the Mediterranean diet (MD) on cancer.

Journal	Count (%)	Citation	*H*-index	IF (2021)	JCR	Country
Nutrients	158 (11.17)	4,927	37	6.706	Q1	Switzerland
British Journal of Nutrition	44 (3.11)	1,795	23	4.125	Q3	England
European Journal of Nutrition	41 (2.90)	801	17	4.865	Q2	Germany
American Journal of Clinical Nutrition	34 (2.40)	1,716	23	8.472	Q1	USA
Journal of Nutrition	33 (2.33)	1,798	21	4.687	Q2	USA
International Journal of Cancer	32 (2.26)	1,187	18	7.316	Q1	Switzerland
International Journal of Molecular Sciences	25 (1.77)	1,748	18	6.208	Q1	Switzerland
PLoS One	24 (1.70)	816	16	3.752	Q2	USA
Nutrition and Cancer-An International Journal	20 (1.41)	282	10	2.816	Q3	USA
European Journal of Clinical Nutrition	18 (1.27)	830	14	4.884	Q2	England

### 3.4. Authors and co-cited authors

**FIGURE 6:**
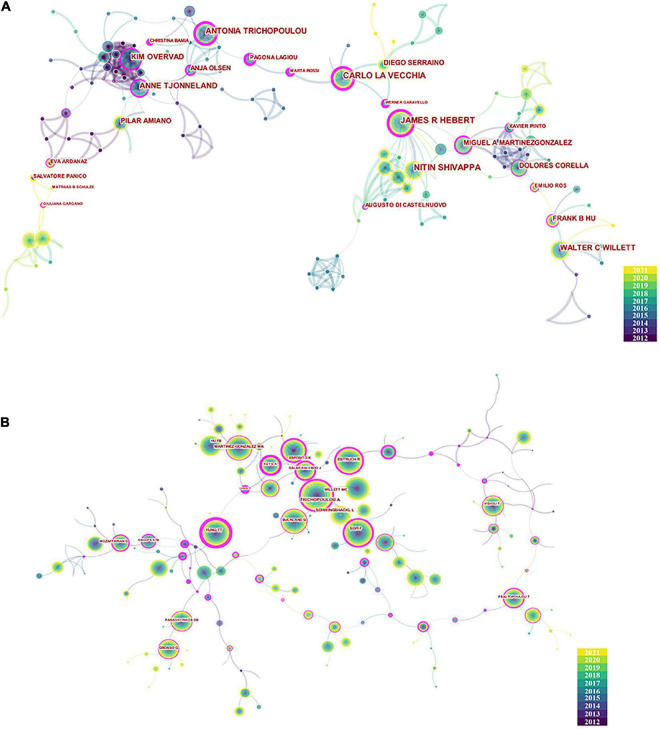
The collaboration map of panel **(A)** authors and **(B)** co-cited authors.

In all, 7,275 authors contributed to the literature on the MD and cancer. The top 10 authors by number of publications and citations are listed in Table 3. Based on the number of publications, the top five authors were James R. Hebert (27), Carlo La Vecchia (24), Nitin Shivappa (24), Antonia Trichopoulou (21), and Anne Tjonneland (19). As shown by the co-authorship network (Figure 6A), many scholars had a centrality greater than 0.1 (purple outer ring), with James R. Hebert (0.54) having the highest centrality, followed by Carlo La Vecchia (0.50) and Antonia Trichopoulou (0.49). These data reveal that these scholars‘ studies serve as a pivot point for the various fields studying the MD and cancer. In terms of co-citations (Table 1), Antonia Trichopoulou had the most citations, reaching 501. Ramon Estruch (310) was second, followed by Francesco Sofi (307), Lukas Schwingshackl (303), and Miguel A. Martinez Gonzalez (271). If two articles were cited by the same document, a co-citation relationship existed between the two articles (29). Figure 6B displays the network of connections between co-cited authors, with the top three centrality scholars including Teresa T. Fung (0.82), Antonia Trichopoulou (0.53), and Frank B. Hu (0.52).

**TABLE 3:** Top 10 productive authors and top 10 co-cited authors related to the Mediterranean diet (MD) on cancer.

Rank	Authors	Count	Centrality	Co-cited author	Citation	Centrality
1	James R. Hebert	27	0.54	Antonia Trichopoulou	501	0.53
2	Carlo La Vecchia	24	0.50	Ramon Estruch	310	0.49
3	Nitin Shivappa	24	0.01	Francesco Sofi	307	0.49
4	Antonia Trichopoulou	21	0.49	Lukas Schwingshackl	303	0.00
5	Anne Tjonneland	19	0.22	Miguel A. Martinez Gonzalez	271	0.10
6	Kim Overvad	16	0.22	Teresa T. Fung	264	0.82
7	Walter C. Willett	16	0.08	Genevieve Buckland	225	0.00
8	Miguel A. Martinez Gonzalez	14	0.34	Walter C. Willett	194	0.02
9	Dolores Corella	13	0.16	Frank B. Hu	170	0.52
10	Frank B. Hu	13	0.13	Katherine Esposito	161	0.14

### 3.5. References

**FIGURE 7:**
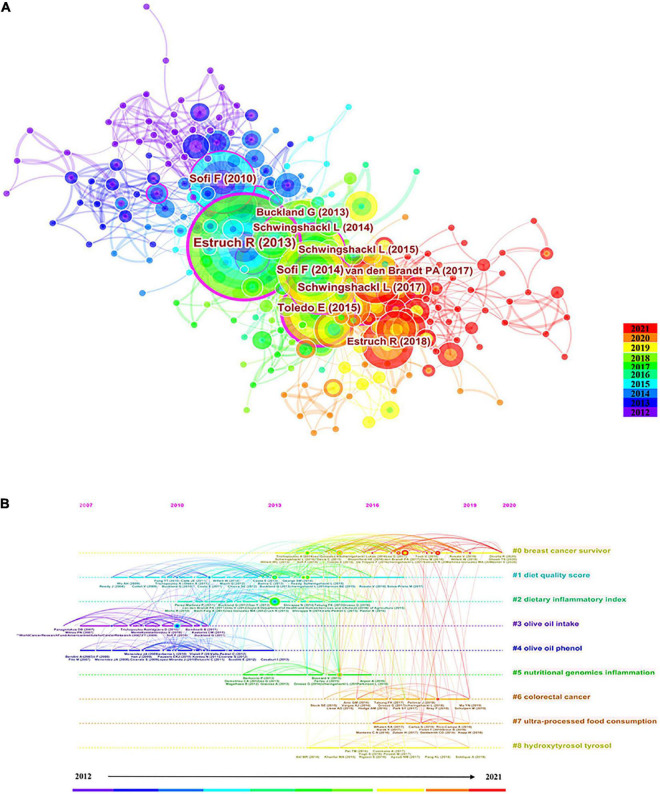
**(A)** The co-occurrence map of references. **(B)** The timeline plot of references. The texts on the right are the topics.

In total, 62,468 references were cited in 1,415 included papers. Table 4 lists the top 10 cited references. The most cited reference was authored by Estruch (30) and had 159 citations, followed by Schwingshackl (31) (88 citations) and Toledo (9) (86 citations). Five of the top 10 cited references had centralities greater than 0.1, with Estefania Toledo’s study having the highest centrality (0.29), followed by Ramón Estruch’s (0.26), and Lukas Schwingshackl’s (0.15). These data show that these references, especially the reference authored by Estefania Toledo, are key points that connect different fields studying the MD and its effects of cancer. The co-citation relationship of these references is presented in Figure 7A. To cluster these references and display temporal trends, a timeline plot was constructed (Figure 7B). In Figure 7B, there are a total of nine clusters that show that the initial research in the area of the MD primarily focused on olive oil (including #3 olive oil intake, #4 olive oil phenol), and that the “diet quality score” was the longest-lasting theme. Additionally, these data showed that BC and CRC were recent research hotspots.

**TABLE 4:** Top 10 high-cited references related to the Mediterranean diet (MD) on cancer.

Rank	References	First Authors	Citation	Centrality	Journal	JCR	IF (2021)
1	Primary prevention of cardiovascular disease with a MD	Estruch et al. (30)	159	0.26	New England Journal of Medicine	Q1	176.077
2	Adherence to MD and risk of cancer: an updated systematic review and meta-analysis	Schwingshackl et al. (31)	88	0.11	Nutrients	Q1	6.706
3	MD and invasive breast cancer (BC) risk among women at high cardiovascular risk in the PREDIMED trial: a randomized clinical trial	Toledo et al. (9)	86	0.29	JAMA Internal Medicine	Q1	44.411
4	Accruing evidence on benefits of adherence to the MD on health: an updated systematic review and meta-analysis	Francesco Sofi (93)	77	0.15	American Journal of Clinical Nutrition	Q1	8.4722
5	MD and health status: an updated meta-analysis and a proposal for a literature-based adherence score	Francesco Sofi (94)	66	0.02	Public Health Nutrition	Q2	4.539
6	Adherence to MD and risk of cancer: an updated systematic review and meta-analysis of observational studies	Schwingshackl and Hoffmann (35)	62	0.15	Cancer Medicine	Q2	4.711
7	Adherence to MD and risk of cancer: a systematic review and meta-analysis of observational studies	Schwingshackl and Hoffmann (36)	61	0.03	International Journal of Cancer	Q1	7.316
8	Primary prevention of cardiovascular disease with a MD supplemented with extra-virgin olive oil or nuts	Estruch et al. (30)	60	0.05	New England Journal of Medicine	Q1	176.077
9	MD adherence and risk of post-menopausal BC: results of a cohort study and meta-analysis	van den Brandt and Schulpen (37)	54	0.09	International Journal of Cancer	Q1	7.316
10	Adherence to the MD and risk of BC in the European prospective investigation into cancer and nutrition cohort study	Buckland et al. (38)	51	0.09	International Journal of Cancer	Q1	7.316

### 3.6. Keywords

**FIGURE 8:**
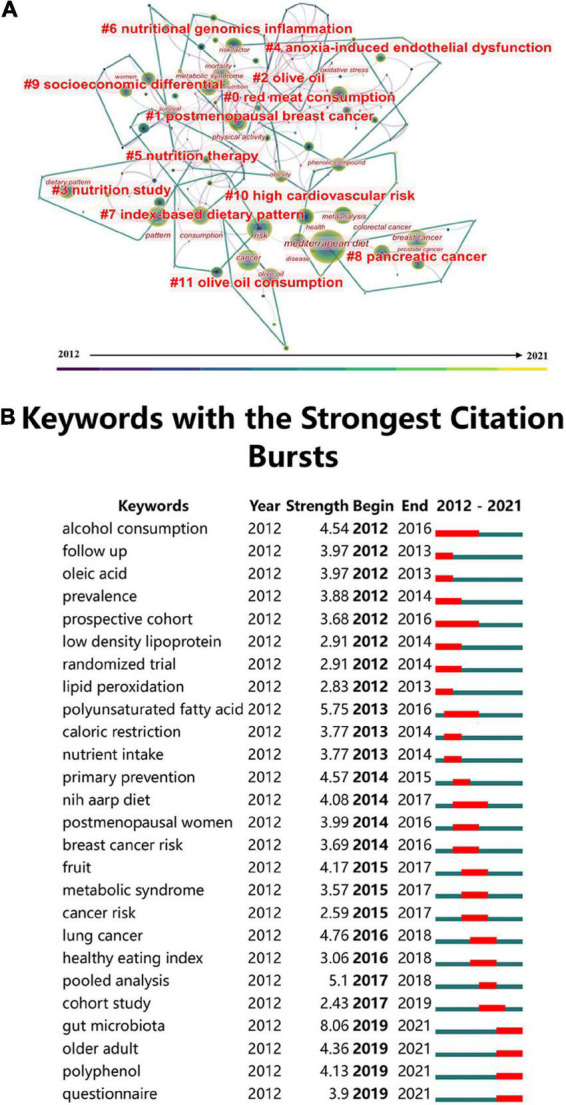
**(A)** Cluster and collaboration map of keywords. **(B)** Bursts detection map of keywords.

Keywords summarize the core material of a paper in great detail. Cluster analysis and co-occurrence analysis of keywords is depicted in Figure 8A. After clustering, a total of 12 clustering labels were produced, which were divided into three main categories: nutrition-related (e.g., #3 nutrition study, #5 nutrition therapy, #6 nutritional genomics inflammation), diet-related (e.g., #0 red meat consumption, #2 olive oil, #7 index-based dietary pattern), and cancer-related (including #1 post-menopausal BC and #8 pancreatic cancer) keywords. Notably, cancer-related keywords such as CRC and prostate cancer were also included in cluster eight. Since 2012, a total of 26 keywords have undergone a sudden burst. The results of burst detection are shown in Figure 8B. Keywords like gut microbiota, older adult, and polyphenol have increased since 2019, proving that research in these keyword-related areas is currently a hot topic and represent future developmental trends in the field of the MD and cancer research. The burst strength of the gut microbiota was 8.06, the highest among the burst keywords. Thus, research in the area of the gut microbiota in relation to the MD and cancer deserves strong attention.

## 4. Discussion

Research on the MD and cancer has increased over the past decade (2012–2021), with the total number of studies exceeding 200 (219) in 2021. This is more than three times that of 2012 and is partly due to cancer already being a focus and hotspot of interest (32). Another more significant reason that research on the MD and cancer has increased in recent years is due to the recently discovered beneficial effects of diet on cancer (33). The rise in studies confirms that the field of the MD on cancer has a promising prospect and requires more attention and in-depth study.

Italy is the country with the most publications in this research area, and together with Spain, Greece, France, and other southern European countries on the Mediterranean coast, it leads the forefront of research on the MD in the cancer field in the world. This is not surprising given its geographical location and dietary habits. In addition to Italy, only the United States has published more than 400 articles on the MD and cancer, which may be a result of the favorable acceptance of the MD and strong scientific research strength in the United States (34). Among scientific research institutions, Harvard University in the United States had the most publications worldwide, which also shows that the United States has a strong scientific research base in the field of the MD and cancer. The majority of the top 10 institutions conducting research on the MD and cancer were located in Italy and Spain, which is consistent with national publishing numbers and trends. In terms of cooperation, Italy and the United States had the highest levels of reciprocal cooperation, independent of whether Italy, Spain, Greece, or France were used as the representatives of the MD countries. The United States, the United Kingdom, Germany, and other countries also showed international cooperation. Collectively, these data suggest that research on the MD and cancer is a field of global cooperation. In addition, some countries/regions need to strengthen international exchanges and cooperation.

*Nutrients* published the most articles in the field of the MD and cancer and was the only journal to include more than 10% of the total of all published research in the area. In addition to this, *Nutrients* ranked first in terms of total citations and *H*-index, indicating that *Nutrients* included a large number of high-quality articles. In conclusion, *Nutrients* is the most authoritative journal on the MD and cancer as a Q1 nutrition journal. The majority of the top 10 journals were nutrition-related, however, a small number of journals in the fields of oncology, biology, and comprehensive journals also published research on the MD and cancer. Research centers in the field were mainly focused on nutrition, and it is foreseeable that more oncology research will appear in the future. According to the dual-map overlap of journals and disciplines, it was found that the current research is mostly centered on Medicine/Medical/Clinical subjects and mainly based on Molecular/Biology/Genetic subjects. Furthermore, the clinical transformation of basic research represents the current research state and trend. In addition, research in the Molecular/Biology/Immunology fields were inseparable from the contributions from the Molecular/Biology/Genetic fields. Some basis of clinical transformation comes from Health/Nursing/Medicine, suggesting that Health/Nursing is also an important source and direction for clinical transformation.

The number of articles published by the author was used in the current study to represent the contribution of the author to the research field, and the number of citations of the co-cited author reflected the influence of the author. The authors Antonia Trichopoulou, Miguel A. Martinez Gonzalez, and Frank B. Hu were within the top 10 contributing authors, both in terms of the number of publications and number of citations. These data indicate that the scientific output and influence of these authors are wide-reaching. Among the top 10 highly cited references, Ramón Estruch, Francesco Sofi, and Lukas Schwingshackl all authored more than two articles. These three authors have all been cited more than 300 times, ranking them among the top five co-cited authors. The most cited reference was published in the *New England Journal of Medicine* (NEJM) and was authored by Ramón Estruch. It was a randomized controlled trial (RCT) that confirmed that the MD reduces the incidence of adverse cardiovascular events in people with high cardiovascular disease risk (30). As an RCT study published in a high-impact journal, with 159 citations, this speaks volumes about the extremely high quality of the study. This study provided further evidence of the cardiovascular benefits of the MD, making its high rate of citation by MD-related research understandable. References related to the MD and cancer mainly included meta-analyses (31, 35–37). Notably, of these meta-analyses, the most cited study (88 citations) was written by Lukas Schwingshackl and was published in *Nutrients*. This meta-analysis was conducted on the adherence of subjects to the MD and their risk of cancer and evaluated 83 studies and 2,130,753 subjects. The results of the study confirmed that higher MD adherence was strongly associated with lower cancer mortality and lower risk of CRC, BC, gastric cancer (GC), liver cancer, head and neck cancer, and prostate cancer. The study published in 2017 was more recently published compared with other references but had a large number of citations, indicating that the study had a strong focus on the MD and cancer and that it was a high quality article. Within the cancer references, a large emphasis was on BC (9, 37, 38). For instance, an RCT study conducted by Estefania Toledo and published in *JAMA Internal Medicine* confirmed the positive significance of the MD on BC in women with high cardiovascular risk. This article also had the highest centrality (0.29), likely because it discussed the effects of the MD on both cardiovascular disease and cancer. The timeline plot visually depicts the development of each MD topic based on references. As a representative of the MD, the intake of olive oil (39, 40), the primary active ingredient of olive oil, and olive oil phenols (41, 42), were the foci of early research. Olive oil phenols exert anticancer effects by inhibiting the proliferation of cancer cell lines that represent leukemia, CRC, and BC and by promoting apoptosis of cancer cells. Recent investigations have focused on hydroxytyrosol, oleocanthal, and other major components in olive oil polyphenols. Hydroxytyrosol as an important phenolic compound unique to olive oil that exerts chemopreventive and treatment effects in cancer. Hydroxytyrosol is a strong antioxidant that reduces the risk of cancer by reducing oxidative DNA damage. Oleocanthal inhibits the growth, proliferation, migration, and angiogenesis of cancer cells, such as those in melanoma, BC, liver cancer, and colon cancer. Oleocanthal also induces apoptosis of tumor cells by increasing reactive oxygen species generation (43, 44). BC is a persistent and hot topic in cancer, and the beneficial influence of the MD on BC is definite, as the MD has been showed to be effective as a means of primary prevention for BC and to lower the incidence of BC (45, 46). Another cancer-related topic covers digestive system tumors and is represented by CRC. The MD is negatively correlated with the risk of CRC and reduces the incidence of CRC (47, 48). The MD is a protective factor for GC and esophageal cancer, and higher MD adherence is linked with a lower incidence of GC and esophageal cancer (49–52).

Keywords can summarize the characteristics and focus of a research field. Clustering results of keywords are comparable to those for references. In addition to nutrition and diet-related clustering, the cancer-related cluster pointed to a protective effect of the MD on pancreatic cancer and prostate cancer (53, 54). Current cancer research frontiers and hotspots were identified by keyword burst detection. The strongest keyword identified was the gut microbiota, which has been a hotspot since 2019. Interestingly, the MD has been shown to cause changes in the gut microbiome (55–57). Regulating the gut microbiota and its corresponding metabolites enhances the effects of anti-tumor therapies and further helps in the investigation of new tumor prevention strategies (58, 59). Such a promising and potential field has inspired researchers, leading to the hypothesis that the MD can prevent and inhibit the development of CRC by regulating the gut microbiota, by maintaining intestinal barrier function, and by reducing inflammation. The MD may increase anti-inflammatory microbiota and simultaneously inhibit pro-inflammatory microbiota that alter intestinal barrier function to improve the dysregulated intestinal ecology (60, 61). Another significant keyword identified in the current study was polyphenols. Olive oil polyphenols are the primary active ingredients of olive oil, whose consumption is encouraged in the MD. Research on olive oil polyphenols has expanded our understanding of their beneficial effects on cancer. The beneficial impact of the MD on tumors may be due to the interaction between olive oil polyphenols and the gut microbiota. For example, olive oil polyphenols can regulate the composition and activity of intestinal microbiota, which transforms the intestinal microbiome to include more protective bacteria. These gut microbiota can produce active metabolites with additional chemopreventive effects through the degradation of polyphenols and other components in olive oil (62, 63).

The MD is a healthy dietary pattern, and cancer is an important factor affecting the security of global public health. The publication of 1,415 related studies in the past decade and the ongoing rise of research in this area have proved the necessity and significance of the MD in cancer research. The current research field illustrates the importance of international cooperation. At present, the field is more focused on the benefits connected to BC. With the support of RCTs and meta-analysis, a high degree of evidence for implementing the MD in the prevention of BC exists. Additionally, the MD has been shown to be beneficial for gastrointestinal cancers, such as CRC, GC, esophageal cancer, and pancreatic cancer. However, the level of evidence for implementing the MD diet for these cancers is relatively low. In the future, more high-quality, multi-center, rigorously designed, high-level, evidence-based medical research is needed to improve the credibility of these research conclusions. Lung cancer and prostate cancer-related research utilizing the MD also exists. However, these non-digestive systems cancer need further attention and in-depth study. Currently, study of the MD and its effects on the gut microbiota is the most popular research direction. The beneficial effects of the MD on tumors is closely related to the gut microbiota, and CRC, specifically in the intestine, has drawn the most attention. In addition to studying the effects of MD on CRC, scholars should put effort into studying the potential benefits of the MD on other gastrointestinal tumors, including esophageal cancer, GC, and pancreatic cancer *via* modulation of the gut microbiota in the future. Research on the primary components of the MD, such as olive oil and polyphenols, particularly hydroxytyrosol, and their mechanisms of action in the prevention and treatment of cancer have been studied. There are relatively a large number of studies on polyphenols, which are of great promise and significance (64). However, the beneficial effects of the MD, either due to a single component or as a synergistic effect of multiple nutrients, is controversial. Olive oil is beneficial to CRC with its effects being opposite to those of red meat. For example, when red meat consumption is used as a keyword cluster, high intakes of red meat and processed meat, and alcohol have been shown to increase the risk of CRC (65). Conversely, increased intake of dietary fiber lowers the risk of CRC (66). High dietary fiber intake is also a part of the MD. According to some researchers, obesity and cancer are related (67), and the healthy MD greatly reduces the probability of obesity thereby preventing some cancers. These studies have indicated that the mechanism by which the MD regulates cancer must be the result of numerous factors, so the identification of the key components of its effects on different cancers and a further in-depth grasp of the molecular mechanisms important for the beneficial effects of the MD is imperative (68). The role of the MD on tumors is mainly reflected in cancer prevention, preventing the occurrence of cancer at its root is the most effective measure to reduce the burden of cancer, which is similar to the old Chinese proverb, “Excellent doctor preventive treatment of disease.”

For advanced cancer, anti-tumor treatments that utilize immunotherapy has become more prevalent and significant (69). The MD is considered a feasible intervention to enhance the effectiveness of immunotherapy by modulating the gut microbiota (70, 71). Therefore, the MD has great potential as an adjunct to immunotherapy. In addition to prevention, the interaction of the MD with anti-tumor therapy may improve the quality of life and promote recovery after surgery. These potential effects and interactions of the MD with other anti-tumor treatments require further research.

The MD is popular with students, adults, and the elderly (72–74). The extensive research of the MD in the field of cancer demonstrates its enormous potential for both cancer prevention and treatment. In addition to the MD, many other dietary patterns may be beneficial for cancer. The Dietary Approaches to Stop Hypertension (DASH) diet originated from a large-scale hypertension prevention and treatment program launched in the United States in 1997. The DASH diet has been found to be effective not only in preventing high blood pressure but also in reducing cancer risk. Studies have confirmed that the DASH diet can significantly reduce the risk of CRC, BC, colorectal adenoma, and other cancers (75–77). The ketogenic diet is a dietary pattern characterized by high fat, low protein, and very low carbohydrate intake. The ketogenic diet promotes metabolism of fats and produces ketones, such as acetoacetate and β-hydroxybutyrate, which may exhibit anti-tumor activities by inhibiting the aerobic glycolytic metabolic pathways in cancer cells (78–80). Numerous studies have also confirmed that the ketogenic diet enhances the therapeutic effects of chemotherapy and radiotherapy and reduces the side effects of chemotherapy and radiotherapy in BC, lung cancer, pancreatic cancer, and others (81–84). The relationship between fasting, such as can be found as part of intermittent fasting and in fasting-mimicking diets (FMDs), and cancer has attracted much attention. Fasting affects the living environment of cancer cells by reducing levels of insulin, blood glucose, etc., and improves the clinical outcome of patients with cancer. Fasting also increases the immunity of normal cells and activates a portion of the immune cell population to improve anti-tumor immunity and lessen the toxic side effects of radiotherapy and chemotherapy (85–87). The mechanisms responsible for the inhibitory effects of fasting on cancers such as CRC and triple-negative BC and the ability of fasting to enhance the anti-tumor effects of chemotherapy have been studied (88–90). Numerous clinical studies have confirmed that a diet that mimics fasting can improve the efficacy of neoadjuvant therapy in patients with HER2-negative BC and improve the quality of life of patients with ovarian cancer and BC who receive chemotherapy (91, 92). This evidence fully illustrates the great potential of dietary patterns represented by the MD for cancer prevention and treatment.

This study is the first to comprehensively summarize and analyze the research foundation, development process, current hotspots, and future trends of the MD on cancer using bibliometrics. The use of CiteSpace, VOSviewer, and R packages to visually analyze results allows researchers to swiftly sort out previous research results and identify hotspots and frontiers in specific research fields of interest. The current study inevitably has intrinsic limitations due to the bibliometric analysis utilized. First, this study only included literature retrieved from the WoS database, as the WoS database provides complete information on research articles and their corresponding citations, both of which are required for bibliometrics. Second, there was a certain degree of language prejudice due to the screening methods used, which allowed only those publications written in English to be included in the analysis. In addition, in terms of search strategy, to optimize the inclusion of pertinent research, this paper adopted a relatively broad topic search. Although a comprehensive search formula has been adopted, some studies may still have been missed.

## 5. Conclusion

Overall, the current study analyzed and summarized the development of the MD and cancer-related research, current hotspots in the field, and future directions. Research on the role of the MD in cancer prevention and treatment is receiving more and more attention. At present, numerous in-depth studies have described the positive effects of the MD on BC, CRC, GC, lung cancer, etc., with research on other cancers being insufficient. Exploration of the mechanisms of interaction between the MD and the gut microbiota, the impact of these interactions on various cancers, and the discovery of the main active ingredients of the MD, mainly represented by olive oil polyphenols, are the current hotspots and future research directions. Scientific advancement is based on the investigation of mechanisms, and clinical trials are intuitive means to confirm efficacy. The effects of the MD on a range of cancers urgently needs more high-quality clinical studies to provide high-level, evidence-based medical evidence and more in-depth molecular mechanistic studies to provide a theoretical basis for the implementation of the MD in preventing and treating various cancers.

## Data availability statement

The original contributions presented in this study are included in the article/supplementary material, further inquiries can be directed to the corresponding author.

## Author contributions

YL was responsible for data collection, data analysis, completing the writing, figures, and tables. JL was responsible for checking data and analysis results and reviewing and revising articles. Both authors contributed to the article, designed the study, and approved the final version of the submitted manuscript.
